# Risk group of AIS is the key to its prophylaxis

**DOI:** 10.1186/1748-7161-10-S1-P10

**Published:** 2015-01-19

**Authors:** Mikhail Dudin, Dmitry Pinchuk, Viktor Pechersky, Tatyana Avaliany, Tatyana Khaymina, Aleksei Shashko

**Affiliations:** 1 Children's Rehabilitation Center of Orthopedics and Traumatology "Ogonyok", Russia; 2 Child’s Orthopedics Center “Rodnik”, Russia; 3 Institute of experimental medicine of the North-West Branch of the Russian Academy of Medical Sciences, St. Petersburg, Russia

## Introduction

One of the fundamental properties of typical AIS is its monomorphism. The C-shaped scoliosis is a “pure” 3D deformation, while S-shaped form consists of two 3D curves, etc. Mathematical modeling has shown a steady sequence of forming units of 3D deformation. Real clinical manifestations in the initial period of development of typical AIS are identical to changes, predicted by mathematical modeling on the basis of identified consistent patterns.

## Objectives

To define the sequence of clinical symptoms and their importance in the transition of a healthy spinal column to "scoliotic" one, that will determine criteria for risk group. It allows us to develop a treatment at the preclinical stage of typical AIS, that is the basis for its prophylaxis.

## Material and methods

During 2012-2013 we observed 600 children of both sexes, aged 9 to 13 years, residing in one settlement. The group included children without signs of AIS. During this period physical and instrumental examination of all these children were carried out every 8-12 months. The instrumental examination included: CDOT, EMG, stabilometry and immunoferment analysis of neuropeptides (oxytocin and arginine-8-vasopressine) level, as posture asymmetry factor.

## Results

**Figure 1:**
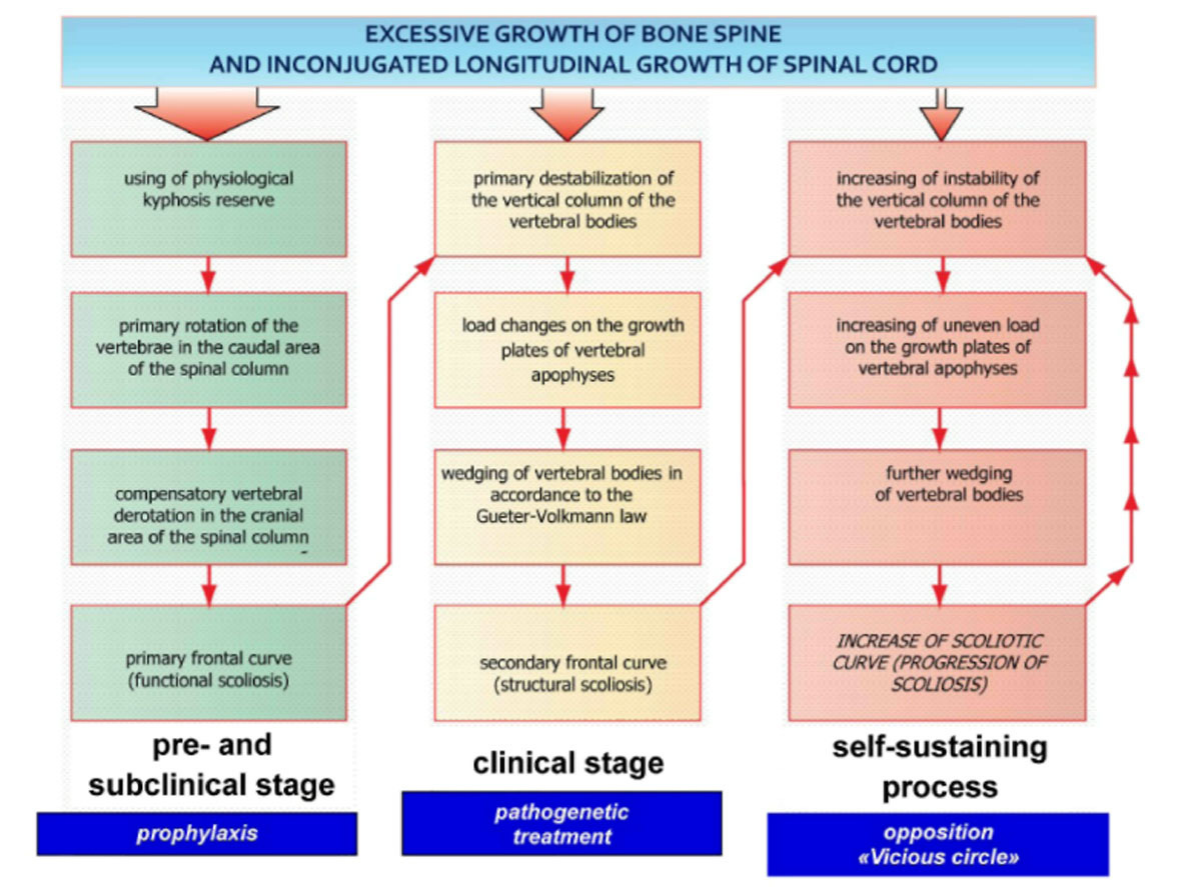
Pathogenesis of AIS

**Figure 2:**
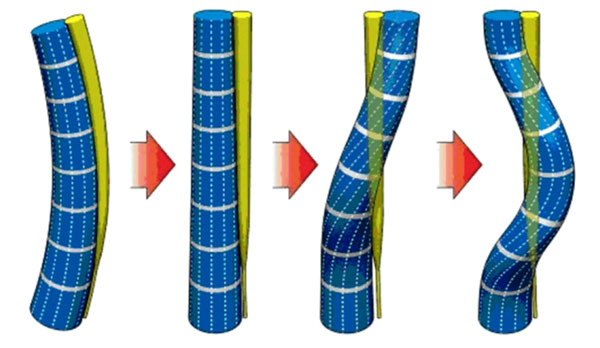
Transformation of healthy spinal column in scoliotic one (sagittal profile)

1. The AIS pathogenesis has been clarified on the basis of obtained data (see Figure 1).

2. The following sequence of clinical symptoms at risk group of typical AIS was defined: normal spine → fat-back (sagittal plane) → flat-back + torsion of all trunk from spine lumbar zone (the first stage of horizontal plane. See Fig. 2). It is the pre-clinical development of the typical AIS (risk group of typical AIS).

But “flat-back + torsion of spine lumbar zone” leads to detorsion of the shoulder girdle or upper part of the trunk (the second stage of horizontal plane). The projection of the spinal canal is straight (not deformed), while in the column of vertebral bodies can be seen two “anticircuits” (opposite direction twisting), which finish the emergence of 3D deformation. It is the beginning of the clinical development of the typical AIS.

3. The obtained data of instrumental examinations were completely identical to described above sequence of clinical symptoms. The greatest interest was aroused by the results of neuropeptides investigation. The altering of their levels was observed even at the end of fat-back formation.

## Conclusion

On the basis of obtained data the complex of therapeutical interventions was created to prevent typical AIS. Currently the clinical testing of this complex is carried out in a representative group of the child population and preliminary results (only for 2013) are encouraging.

